# A solar cycle clock for extreme space weather

**DOI:** 10.1038/s41598-024-58960-5

**Published:** 2024-04-08

**Authors:** S. C. Chapman, T. Dudok de Wit

**Affiliations:** 1 Physics Department, Centre for Fusion, Space and Astrophysics, University of Warwick, Coventry, UK; 2 International Space Science Institute, Bern, Switzerland; 3 Department of Physics and Statistics, University of Tromso, Tromsö, Norway; 4 LPC2E, CNRS, CNES, University of Orléans, Orléans, France

**Keywords:** Solar cycle, Space weather, Magnetospheric physics, Solar physics, Astrophysical plasmas, Natural hazards, Space physics

## Abstract

The variable solar cycle of activity is a long-standing problem in physics. It modulates the overall level of space weather activity at earth, which in turn can have significant societal impact. The Hilbert transform of the sunspot number is used to map the variable length, approximately 11 year Schwabe cycle onto a uniform clock. The clock is used to correlate extreme space weather seen in the *aa* index, the longest continuous geomagnetic record at earth, with the record of solar active region areas and latitudes since 1874. This shows that a clear switch-off of the most extreme space weather events occurs when $>90$% of solar active region areas have moved to within about 15° of the solar equator, from regions of high gradient in solar differential rotation which can power coronal mass ejections, to a region where solar differential rotation is almost constant with latitude. More moderate space weather events which coincide with 27 day solar rotation recurrences in the *aa* index, consistent with stable, persistent source regions of high speed streams, commence when the centroid of solar active region areas moves to within 15° of the solar equator. This offers a physical explanation for the longstanding identification of a two component cycle of activity in the *aa* index.

## Introduction

Extreme space weather storms have the potential for significant societal impact and financial loss^1,2^, disrupting power distribution, communication, aviation and satellites. Statistical estimates of the likelihood of occurrence of extreme space storms is central to decision making for mitigating the effects of space weather which necessarily must balance resilience against cost. Whilst the severity of the technological and societal impact of a geomagnetic storm depends on many factors^3^, from the details of the structure emitted from the corona, its solar wind propagation, to how it interacts with earth’s magnetosphere^4–6^, the most extreme events are directly driven by, and directly correlate with, large-scale solar eruptions^7–9^. Quantifying extreme space weather risk is thus intimately related to understanding the solar cycle of activity, which in turn is a longstanding unsolved problem in physics^4^, informing our understanding of the solar dynamo which is as yet not complete^10^.

**Figure 1:**
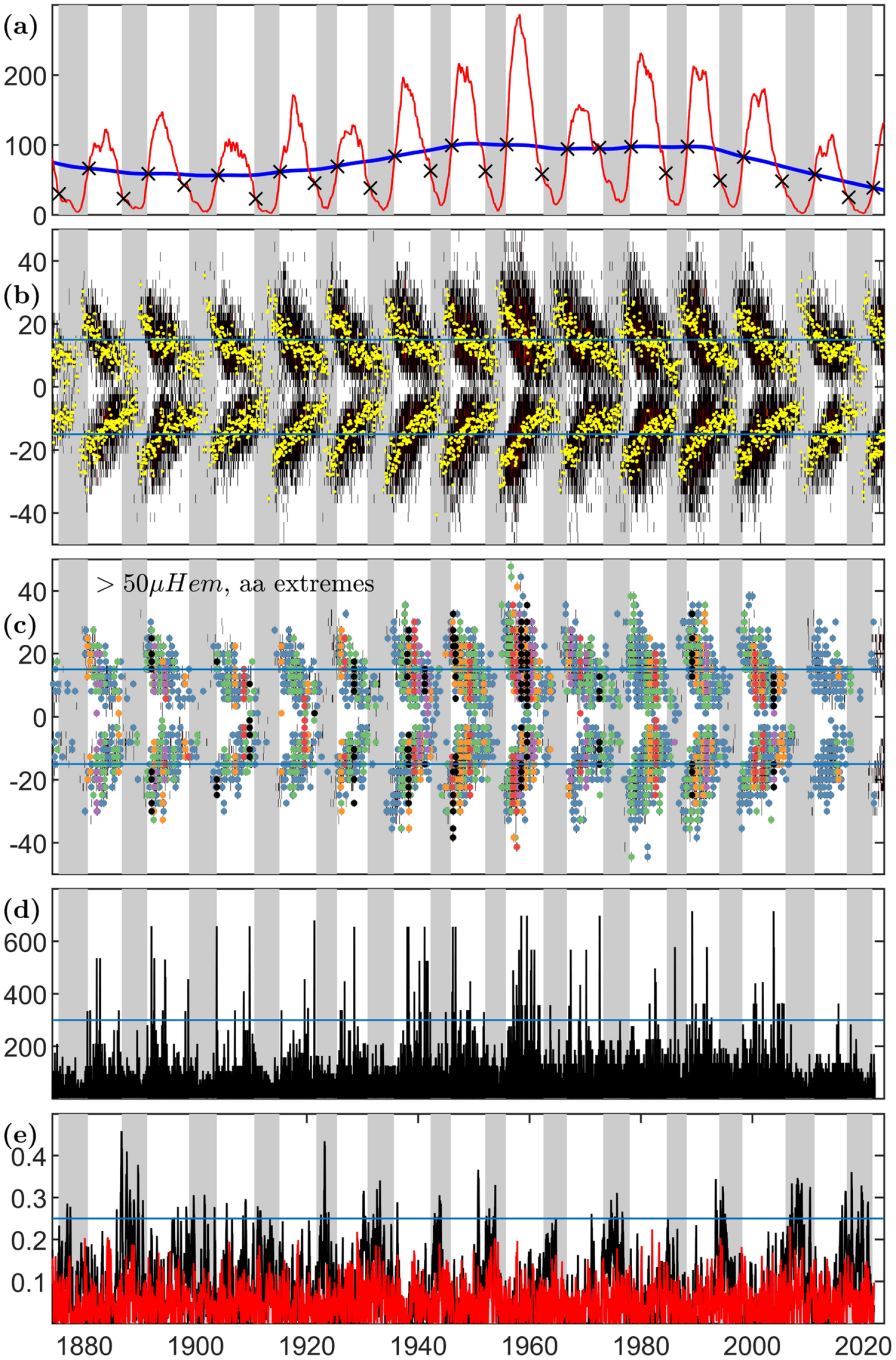
Time evolution of sunspot number, area latitudes and extreme and recurrent geomagnetic activity. Grey shading indicates the quiet interval between switch-off and switch-on on all panels. Panel (**a**): 13 month smoothed SSN (red) and 40 year smoothed SSN (blue). Black crosses are at times 12 months following the smoothed SSN downcrossing of the 40 year smoothed SSN, and at the smoothed SSN upcrossing of the slow trend, providing estimates of the switch-off and switch-on times respectively. Panel (**b**): active region areas in single Carrington rotation-latitude bins (black) overplotted with the area centroid for each Carrington rotation (yellow). Panel (**c**) overplots on all sunspot area latitude bins with area > 50 μ Hem the Carrington rotation during which the *aa* index exceeds a threshold of 100 (blue) 200 (green) 300 (orange) 400 (red) 500 (purple) 600 (black) nT. Horizontal blue lines on panels (**b**,**c**) indicate latitudes $\pm 15^{\circ }$. Panel (**d**): the *aa* geomagnetic index, blue line indicates *aa* = 300 nT. Panel (**e**) *aa* index autocovariance at a lag of 27 days, approximately the solar rotation (black) and at 10 days (red), to provide an indication of the level of ‘by chance’ autocovariance, this is exceeded by the horizontal blue line at 0.25.

Long-term observations over multiple solar cycles are essential to track solar cycle variability since no two cycles are of the same amplitude and duration. Figure 1 plots an overview of the long-term solar and geophysical records (see Methods) that are analysed in this Letter. The approximately 11 year Schwabe cycle is tracked by sunspot number observations for which monthly records are available from 1749. Panel (a) of Fig. 1 plots the 13 month smoothed sunspot number (SSN) for the last 13 Schwabe cycles. Sunspot latitudes or more specifically, the latitudes of solar coronal active regions (ARs)^4^ execute a classic butterfly pattern as shown in Fig. 1b, emerging at mid-latitudes early in the Schwabe cycle and migrating towards the solar equator where their magnetic flux annihilates, as part of the birth of the next cycle. The time interval of our analysis, shown in Fig. 1, begins at the start of the AR record.

Long-term ground based magnetometer observations at earth track the geomagnetic response to the solar cycle of activity^4,7^. The longest continuous record of the magnetic field at earth is provided by the *aa* index^11^ which is plotted in panel (d) of Fig. 1. Overall geomagnetic activity as seen in the *aa* index tracks the solar cycle variation in solar activity^12,13^, and overall levels of geomagnetic activity can be good solar cycle predictors^14,15^. The *aa* index is however highly discretized^16,17^; high fidelity geomagnetic data, available over the last 4–5 cycles confirm that the high quantiles of geomagnetic activity track the variation in solar activity both within and between solar cycles^17–19^.

Although the details are complex, the overall picture of extreme geomagnetic storms is primarily driven by coronal mass ejections (CMEs) during solar cycle maximum, when the ARs are at higher solar latitudes, and by solar co-rotating high speed streams during the declining phase, when the ARs are at lower latitudes^7^. This is reflected in the solar wind state which is more disordered at maximum^20^. The declining phase is characterised by high speed streams in the solar wind^21^ and recurrences in long-term geomagnetic indices on the 27 day solar rotation timescale^22–25^, as can be seen in the autocovariance of the *aa* index at 27 day lag plotted in Panel (e) of Fig. 1. There are subtleties in identifying the coronal sources of CMEs and recurrent high speed streams; ARs and coronal holes are discriminated by coronal magnetograms, available over the last few cycles only^26^. High speed streams may originate from corotating solar equatorial structures or polar coronal holes^21^. Nevertheless, a reasonable working hypothesis for performing statistical correlations over multiple solar cycles is that the latitudes of the ARs provide an overall indicator of a range of latitudes from which CMEs and (non-polar) high speed streams originate.

CME initiation and evolution is complex^27,28^ however the overall source of energy of a ‘normal’ or ‘standard’ CME is understood to be the magnetic energy from strongly sheared magnetic field^29^. The magnetic field is sheared by the differential rotation of the outer convective region of the sun. The axisymmetric differential rotation increases with decreasing latitude, maximising at the solar equator. The differential rotation varies only weakly across these low latitudes, varying in total by about 13% (see Methods) across latitudes $\pm 15^{\circ }$ of the equator^30–32^. Since the meridional flow is 1–2 orders of magnitude weaker^32^, this supports the overall picture that extreme eruptions initiated at mid latitudes are ultimately powered by shearing of magnetic field driven by the latitudinal gradient in differential rotation, corresponding to more frequent and higher energy CMEs, so that CME rates roughly track the SSN^28^ and are less energetic around solar minimum^33^. Conditions around solar maximum then result in the most intense, CME driven geomagnetic activity. CME driven activity will decline as the ARs move to lower latitudes and geomagnetic activity will be more predominately driven by recurrent high speed streams which originate in regions of relatively low latitudinal gradient in differential rotation, either at the poles or at low latitudes, are not predominantly shear-driven, and are thus stable over multiple solar rotations. To see this overall effect in the AR record, panel (c) of Fig. 1 colour codes each latitudinal bin containing a moderate-to large AR area (> 50 μ Hem) with the level of activity in the *aa* geomagnetic index during each Carrington rotation (the panel is reproduced for other AR area thresholds in Figure SI2 which shows the same result). The colours indicate if within each Carrington rotation, *aa* exceeds the levels [100,200,300,400,500,600] nT. The AR record comprises all AR areas binned by latitude and Carrington rotation and so does not discriminate a single specific latitude of the solar eruption which could drive an extreme event seen in the *aa* index at earth. A given event identified by an exceedence in the *aa* index then appears as a vertical line of coloured dots highlighting all latitudinal bins within which the AR area exceeds > 50 μ Hem during the Carrington rotation within which the *aa* exceedence occurred. The horizontal blue lines are at $\pm 15^{\circ }$ and whilst the colour coding indicates *all* latitudes at which there are ARs, the plots show that the most intense events (*aa* > 300 nT, orange, red, purple, black) only occur when there are a subset of ARs at latitudes above about $15^{\circ }$ in either hemisphere, whereas when the ARs are all within $15^{\circ }$ of the equator, the events are less intense (*aa* > 300 nT, blue, green).

AR latitudes then in principle contain information on the likelihood of extreme space weather events over the solar cycle. Exploiting this requires systematic timing of the phase of the solar cycle, which is challenging given that no two cycles are of the same length. Normalizations of the SSN cycle onto a uniform timebase have included collapse of the SSN profile^4,34^ and the average modulus sunspot latitude^34^. The Hilbert transform of the SSN record can be used to map the non- uniform duration solar cycle onto a uniform interval $2 \pi$ of analytic phase^13^, see Methods and Supplemental Figure SI1. This provides a regular clock for the solar cycle which can be used to organise long-term observations of solar and geomagnetic activity. The Hilbert transform analytic phase acts as a single unifying parametric coordinate for different indicators of the state of solar activity across the solar cycle, and corresponding geomagnetic response at earth.

From the solar clock, a clear quiet interval in the solar cycle can be identified^13^ which spans a uniform interval of $\sim 4\pi /5$ in analytic phase, centred on solar minimum. This quiet interval has a duration of of 4.4 years in an exactly 11 year cycle but is non uniform in time in the SSN record; these quiet intervals are indicated with grey shading on Fig. 1. The switch-off and on of activity are identified at phase $\pm 2 \pi /5$ in advance of, and following, the average phase of the cycle minima which have been identified in the SSN record of the last 25 solar cycles. The switch-on of activity at the end of the quiet interval approximately coincides with the average analytic phase over which the last 12 solar cycle terminators have occurred, these terminator times^35–37^, are identified individually from multiple observations of coronal magnetic activity. The cycle termination is observed as an abrupt reduction in the density of EUV bright point density around the solar equator, marking the final cancellation of the old cycle (magnetic) activity bands at the equator^35^, coinciding with the emergence of mid-latitude sunspots of the new solar cycle butterfly pattern.

The switch-off and switch-on of activity which bracket the quiet interval are however obtained solely from the SSN timeseries. Whilst they were originally identified from the SSN Hilbert phase^13^, the switch-off/on can be read directly from the SSN record as indicated by the black crosses on Fig. 1a occurring respectively at approximately 1 year following the downcrossing, and at the upcrossing, by the 13 month smoothed SSN (Fig. 1a red) of the 40 year smoothed SSN (Fig. 1a blue)^38^. The 40 year smoothed SSN tracks the Gleissberg cycle^25,39^.

Figure 1d then points to a physical explanation of the empirical result obtained from constructing the solar clock^13^: extreme geomagnetic storms (*aa* > 300 nT) rarely occur within the quiet interval (grey shaded regions). Whilst the switch-on coincides with the termination of the extended cycle, the switch-off hitherto was only identified in a sharp overall reduction in extreme activity in the *aa* index, and other activity indicators^13,40^. A key result of this Letter is that the switch-off coincides with the ARs migrating to latitudes within approximately $\pm 15^{\circ }$ of the solar equator as can be seen in panels (b,c) of Fig. 1. The switch-off then corresponds to the bands of ARs reaching the vicinity of the solar equator where the differential rotation is weakly varying so that the magnetic shearing driving CMEs is weaker. The switch-on follows the emergence of ARs at mid-latitudes where the differential rotation latitudinal gradient, and hence the magnetic shearing driving CMEs, is strong.

**Figure 2:**
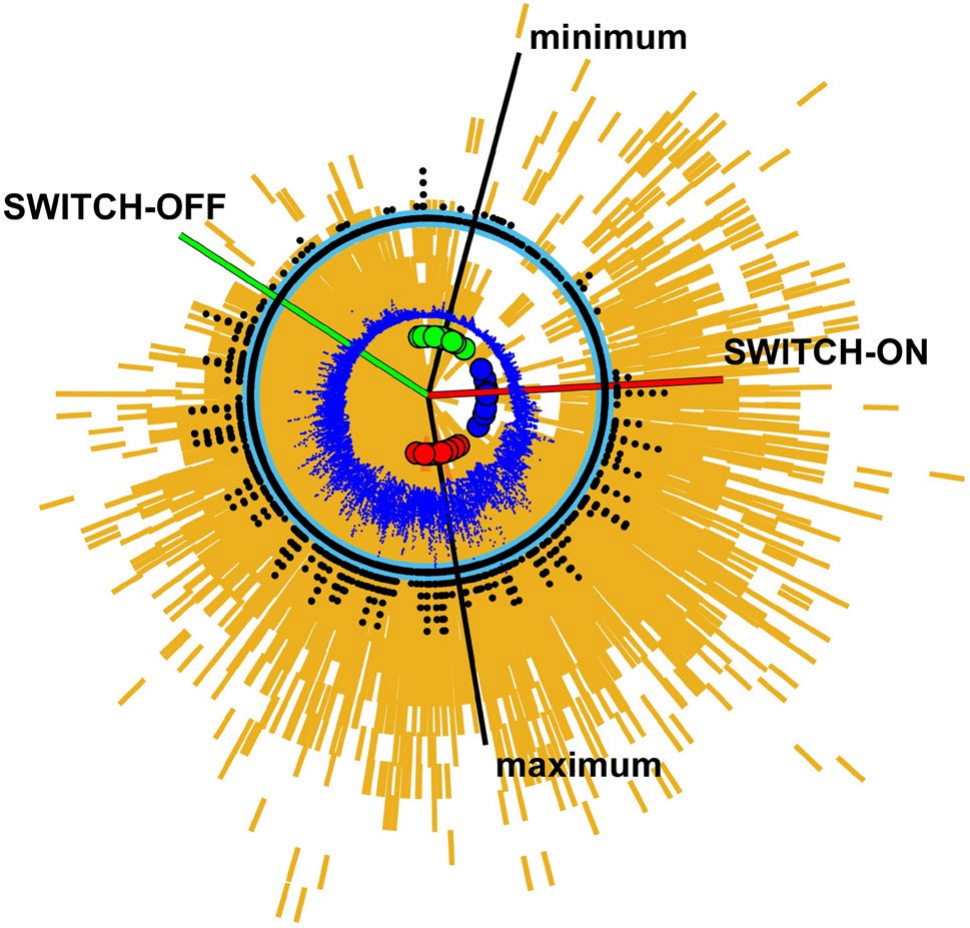
Clock of regular 11 year cycle showing area latitudes and extreme geomagnetic activity. Solar cycle clock constructed such that increasing time (analytic phase) is read clockwise. The analytic phases of the maxima and minima are indicated by red and green circles respectively and the blue circles indicate previously identified terminators^37^. Black lines indicate the average analytic phase for the 25 cycle average maxima and minima. The switch-off (green line) and on (red line) are at $\pm 2 \pi /5$ in phase either side of the 25 solar cycle average minimum phase. Black dots arranged on concentric circles where increasing radius indicates *aa* values which in any given day exceeded 100, 200, 300, 400, 500, 600 nT. Blue dots overplot daily F10.7. Orange overplots all unsigned latitude bins containing AR areas exceeding 50 μ Hem with zero latitude at the clock centre and 15° latitude marked by the blue circle.

Mapping time to SSN Hilbert analytic phase (see Methods) allows us to overlay multiple cycles of activity on a single normalized cycle. This solar cycle clock is constructed in Fig. 2. On the clock, the switch-off (green) and switch-on (red) bracket the $4\pi /5$ quiet interval centred on the average solar mininum. Daily exceedences in the full record of the *aa* index (over 14 solar cycles) are indicated by black ‘spokes’ and these show that extreme events in *aa* rarely occur in the quiet interval. The (unsigned) latitudes of bins containing AR areas > 50 μ Hem for the full AR record (over 13 solar cycles) are shaded orange, latitude is plotted from zero at the centre of the clock, so that as time increases clockwise, the AR latitudes spiral inwards. The blue circle indicates unsigned latitude of $15^{\circ }$. The switch-off then coincides with almost all of the AR migrating to below $15^{\circ }$ in latitude. The AR start to reappear at high latitudes around minimum, and progressively occupy lower latitudes thereafter, reaching $15^{\circ }$ latitude around the switch-on.

**Figure 3:**
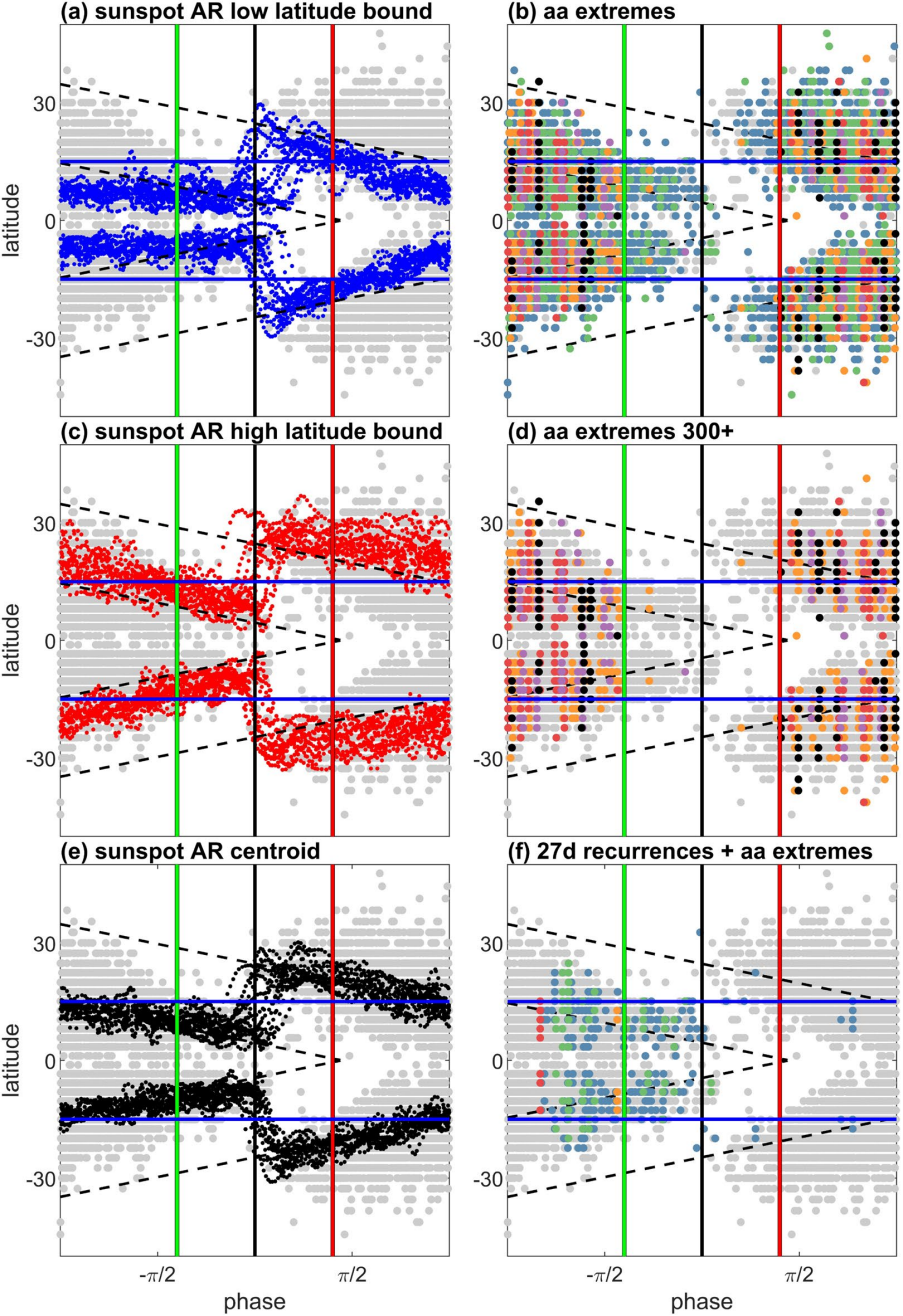
Active region area latitudes and extreme and recurrent geomagnetic activity overplotted for the last 13 solar cycles. All panels: latitude plotted as a function of Hilbert phase of the SSN record (see Methods), grey circles plot centres of latitude bins for each Carrington rotation containing total AR area exceeding 50 μ Hem, horizontal blue lines indicate $\pm 15^{\circ }$ latitude. The 25 cycle averaged minimum (black vertical line) is at phase zero and the switch-off (green line) and on (red line) are at phase $\pm \frac{2\pi }{5}$ either side. Black dashed lines plot the extended cycle estimated from AR latitude centroids (see Methods). Left panels: Lines plot 13 Carrington rotation smoothed latitudes which are the low latitude envelope (90% of total AR area are at higher latitudes, Panel **a**, blue) and the high latitude envelope (90% of total AR area are at lower latitudes, panel **c**, red) and AR area centroid (panel **e**, black). Right Panels: Panel (**b**) overplots on all AR area latitude bins with area > 50 μ Hem the phase of the Carrington rotation during which the *aa* index exceeds a threshold of 100 (blue) 200 (green) 300 (orange) 400 (red) 500 (purple) 600 (black) nT. Panel (**d**) as for panel (**b**), for *aa* index exceeding 300, 400, 500, 600 nT. Panel (**f**) as for panel (**b**) for Carrington rotations where the *aa* index both exceeds the 100–600 nT threshold and has a 27 day lag autocovariance exceeding 0.25.

The relationship between AR latitudes and geomagnetic activity is charted in more detail in Fig. 3. The panels in Fig. 3 plot the solar clock of Fig. 2 unwrapped so that AR latitude is plotted versus solar cycle phase, with the averaged solar minimum at the centre of each panel (black vertical line) and the switch-off (green line) and switch-on (red line) at $-2 \pi /5$ and $+ 2 \pi /5$ respectively. All panels overplot for the full AR database (over 13 solar cycles) the latitude bins and Carrington rotations containing significant (ie > 50 μ Hem) AR areas in grey. Blue horizontal lines indicate $\pm 15^{\circ }$ latitude. The left-hand panels summarize the latitudinal extent of the ARs. The low and high latitude boundaries of the AR areas in each Carrington rotation are shown in panels (a) and (c). These panels plot the 13 Carrington rotation smoothed latitudes above (a, blue) and below (c, red) which 90% of the AR area is found during each Carrington rotation. Panel (e, black) plots the smoothed AR latitude centroid of each Carrington rotation. For all 13 cycles of AR data, panel (c) shows that the switch-off marks the time when at least 90% of the AR have moved to within approximately 15° of the equator. The switch-on coincides with the AR extending from $\pm \; 15^{\circ }$ to higher latitudes, so that their low latitude boundary, the blue lines in panel (a) just extends to $\pm 15^{\circ }$, and their centroids, the black lines in panel (e) are within $\pm 30^{\circ }$ of the solar equator. This is consistent with the observed latitude distribution of halo CMEs^41^, that is, the subset of CMEs that are directed toward earth and are potentially geoeffective.

The AR area data does not allow us to pin-point which sunspot group, recorded as a binned AR area, is the genesis of an extreme solar eruption. However we can for each Carrington rotation during which an extreme event is seen in the *aa* index indicate which latitude bins contain significant (ie > 50 μ Hem) sunspot areas. This is shown in panels (b) and (d) which show that the more extreme events $aa$> 300 nT very rarely occur within the quiet interval. The extreme events switch-off when the ARs are within about 15° of the equator. This can also be seen in each individual cycle which are plotted in Supplemental Figure SI4. The extreme event switch-on is when the AR areas extend from approximately $\pm 15^{\circ }$ to higher latitudes so that the most active, shear dominated latitudes are fully populated with sunspot activity.

If we hypothesise that the switch off of the most extreme events seen in the *aa* index is due to sunspot AR areas moving to a region of reduced gradient in overall solar differential rotation, then this should mark the transition from shear dominated extreme events (CMEs) to high speed streams. This is shown in Fig. 3 panel (f) which indicates the latitude bins containing significant AR areas during Carrington rotations where there is both extreme *aa* activity and significant 27 day autocovariance. This corotating high speed stream associated activity is first seen when the sunspot centroids (panel (e)) move to within 15° of the equator, so that the AR areas are spanning $\pm 15^{\circ }$ (see also the individual cycles plotted in Fig. SI4). It corresponds to an interval of more moderate events, *aa* typically less than 300*nT* which commences before, and overlaps with, the switch-off and continues until solar miminum, when ARs start to reappear at high latitudes.

Although the AR appear in a highly intermittent manner, when their overall latitudinal path is tracked, it forms part of an extended cycle of activity^42,43^ which commences at high latitudes and terminates at the equator^35^. The extended cycle latitudinal bands can be identified in multiple features of activity (^43,44^ and refs. therein). However, a simple model for the track of the extended cycle latitudes can be obtained solely from the AR timeseries (see Methods, and Fig. SI3) and this is plotted with black dashed lines on all panels of Fig. 3. The simple model is obtained from fitting a linear relationship between AR area centroid latitudes seen in the more active half of the cycle, and Hilbert phase. This extrapolates to zero latitude approximately at the switch-on, so that it roughly coincides with the average of terminator times that have been directly identified as the time when EUV bright point annihilate at the equator^37^. The AR area latitudes for the active sun then directly relate to/predict the cycle termination and birth of the new cycle. The extended cycle track crosses $\pm 15^{\circ }$ latitude at phase $\pm \pi$, that is, half a normalized Schwabe cycle in advance of/following solar minimum (which is not at solar maximum, see Fig. 2).

**Figure 4:**
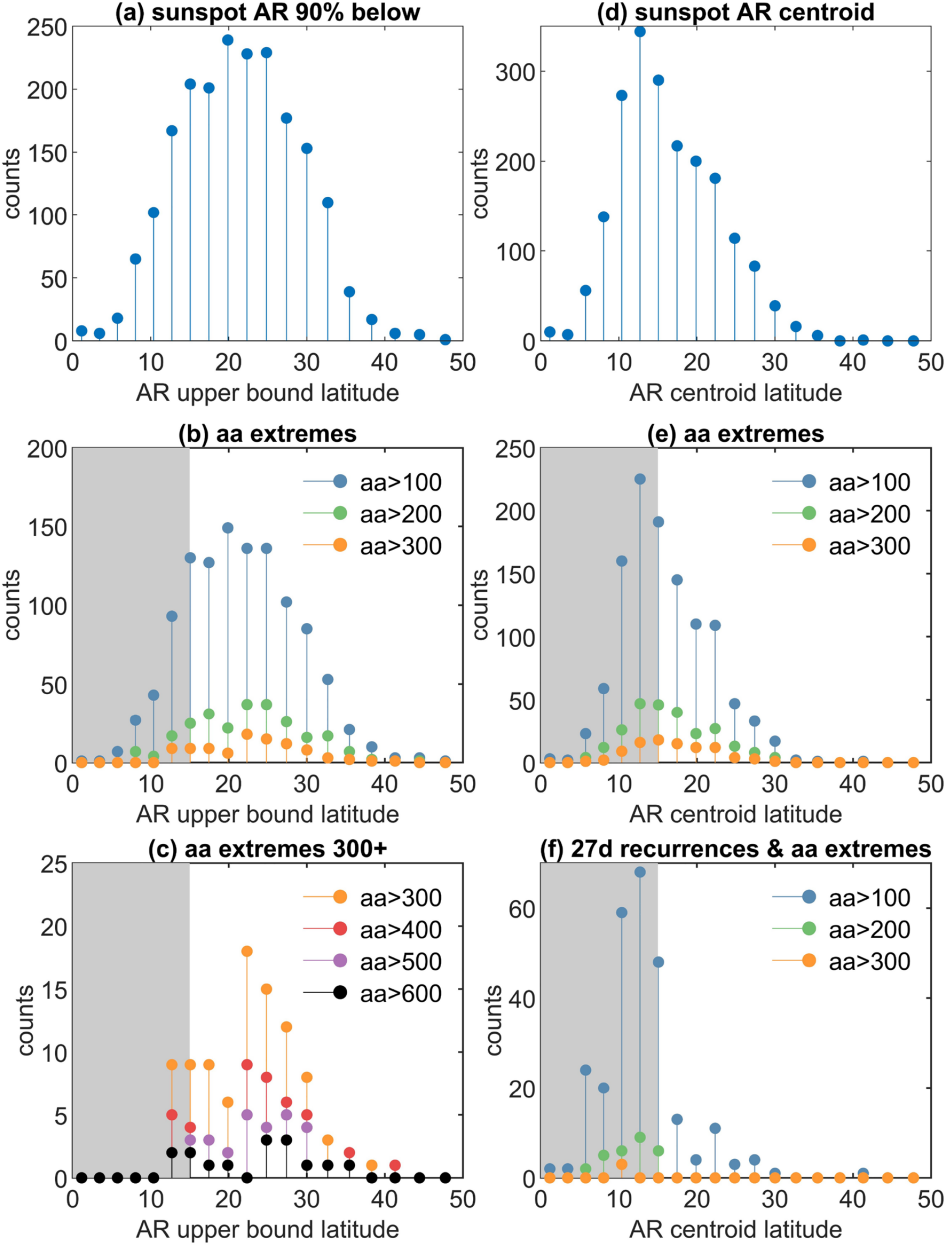
Histograms of active region area latitudes and extreme and recurrent geomagnetic activity for the last 13 solar cycles. Panels plot the histograms of occurrences of the modulus extremum of active region latitudes in each Carrington rotation at the bin centres of uniform bins in sin(latitude) as in the AR dataset. Panel (**a**) plots the high latitude upper bound enclosing 90% of the active region areas in each Carrington rotation. Panel (**b**) plots the subset of the latitude upper bounds binned in (**a**) for Carrington rotations during which the *aa* index exceeds a threshold of 100 (blue) 200 (green) 300 (orange) nT. Panel (**c**) as for panel (**b**) but plots the subset of the latitude upper bounds for Carrington rotations during which the *aa* index exceeds a threshold of 300 (orange) 400 (red) 500 (purple) 600 (black) nT. Panel (**d**) plots the latitude of the AR centroid in each Carrington rotation. Panel (**e**) plots the subset of the centroid latitudes binned in (**d**) for Carrington rotations during which the *aa* index exceeds a threshold of 100 (blue) 200 (green) 300 (orange) nT. Panel (**f**) plots the subset of the centroid latitudes binned in (**d**) for Carrington rotations during which the *aa* index both exceeds a threshold of 100 (blue) 200 (green) 300 (orange) nT and has a 27 day lag autocovariance exceeding 0.25. In all panels grey shading indicates latitudes less than $\pm 15^{\circ }$.

The information in Fig. 3 is displayed in histogram form in Fig. 4. For each Carrington rotation we have identified the upper bound latitude within which 90% of the AR areas are located. Figure 4a is a histogram of the upper bound or modulus extremum, so that if 90% of the AR areas lie within latitudes $+x$ and $-y$ above and below the solar equator, this modulus extremum is $max(|x|,|-y|)$. Figure 4c overplots histograms of these latitudes for Carrington rotations when the *aa* index exceeds 300, 400, 500, 600 nT. These extreme events do not occur when 90% of the AR areas are located within $12-15^{\circ }$ latitude of the solar equator. For each Carrington rotation we have also identified the AR areas centroids, and a histogram of these is shown in panel (d). Panel (f) of Fig. 4 then overplots histograms of these centroid latitudes for Carrington rotations when both the *aa* index exceeds 100, 200, 300 nT and its autocovarance shows a 27 day recurrence. These solar rotation recurrent associated events occur at a much higher frequency once the AR area centroids are within 15° of the solar equator.

In summary, our main result is that the most extreme geomagnetic storms tend to ‘switch-off’ when over 90% of the active regions are located within about 15° latitude of the solar equator. More moderate to extreme events which correlate with a 27 day recurrence in the *aa* index, commence when the centroid of the active regions is within about 15° latitude of the solar equator. We then hypothesise that this correlates with the known latitudinal variation of solar differential rotation. It suggests a scenario in which the most extreme events, which arise from coronal mass ejections powered by the differential rotation gradient, only ‘switch-off’ when almost all of the active regions are within 15 degrees of the equator, where solar differential rotation is almost constant with latitude. Persistent high speed streams, which are seen in 27 day recurrences of the *aa* index, start to drive events when a significant proportion of the active regions are at these lower latitudes. Taken together, these results highlight the importance of considering the latitudinal distribution of the active regions, and not just the active region total area, or the sunspot number.

This scenario does not exclude the occurrence of extreme events when the AR areas are at low latitudes, rather it suggests a mechanism for the observed modulation in their likelihood of occurrence^13^. Across the full *aa* record there is one extreme, $aa$> 300 nT event, in 1986, that occurred in the quiet interval between the switch-off and on (which can be seen in Fig. 2 at just before ‘12 o’clock’). Intriguingly, Fig. 2 (see also Figure 5 of^38^) also shows that extreme events have occurred just after the switch-on and just before the switch-off. Not all events occurring close to the switch off correlate with AR areas above 15 degrees latitude, for example the 1921 event has been identified with a large low-latitude spot group^45^ which can also be seen in panel (c) of Fig. 1. Further work that identifies the detailed conditions of the source regions of extreme events, such as specific active region latitudes, areas and spacing, and the local magnetic and flow conditions, may provide more insight into the physical processes underlying the switch-on and off.

Extreme space storms are rare, so that statistical quantification of the solar cycle variation of their occurrence probability is challenging^46^. It is intimately related to a quantitative understanding of the solar cycle of activity. The Hilbert transform of the 13 month smoothed SSN can be used to map the variable length Schwabe cycle of activity onto a uniform clock. This reveals a clear correspondence between active region latitudes and both the level, and class, of extreme geomagnetic activity at earth. Although it is well understood that there is an overall relationship between the butterfly pattern of sunspot activity with solar latitude, the SSN, and geomagnetic activity at earth, the solar cycle clock pin-points the phase in the cycle when the most extreme, CME driven events are switched-off as their potential AR area sources move to within about 15° of the equator, where the latitudinal gradient in differential rotation is reduced. This overlaps with the onset of more moderate events that are coincident with 27 day recurrences in the *aa* index, consistent with high-speed streams. This directly relates the latitude of the sunspot ARs, and the track of the extended solar cycle, with the longstanding identification by Feynman^14^ (see also^15,47^) of a two-component cycle of geomagnetic activity with two different sources-one due to solar activity (flares, CMEs, and filament eruptions) that follows the sunspot cycle and another due to recurrent high speed solar wind streams that peaks during the decline of each cycle.

The pattern of activity revealed by the solar cycle clock provides a metric for systematic comparison with dynamo theories of the solar cycle. Model predictions of SSN can be translated into a clock which specifies the corresponding AR overall latitudes and the track of the extended cycle. Given that Feynman’s interplanetary component of the *aa* index is a good predictor of the strength of the following solar cycle^14,15^, using the clock to organise model-data comparison may also inform solar cycle prediction.

## Methods

### Construction of the solar clock

The analytic signal^48,49^ $A(t) exp[i \phi (t)]=S(t) +i H(t)$ with time-varying amplitude *A*(*t*) and phase $\phi (t)$ was obtained for the monthly total sunspot number (SSN) time series *S*(*t*) and its Hilbert transform *H*(*t*). This provides a mapping between time and signal phase, converting the (variable) duration of each solar cycle into a corresponding uniform phase interval, from 0 to $2\pi$. A standard method was used to obtain the discrete analytic signal^50^ which satisfies both invertability and orthogonality. The analytic signal will only be physically meaningful if the instantaneous frequency $\omega (t) =d\phi (t)/dt$ remains positive^49^. This is ensured by removing both fast fluctuations and a slow trend before performing the Hilbert transform (see e.g.^13,49^). The slow trend was obtained by a robust local linear regression of the monthly SSN which down-weights outliers (‘rlowess’) using a 40 year window, which essentially obtains the Gleissberg cycle^25^. The Hilbert transform was the performed on a 13 month moving average of the SSN, with this slow trend subtracted, as detailed in Supplemental Fig. SI1. The absolute signal phase is arbitrary and here is set to zero at the average of the phases of the SILSO mimima of cycles 1–25. The location of the switch-off and on are then set at phases $\pm 2 \pi /5$ either side of zero. The switch-on closely coincides with the average phase of the last 12 terminators^37^, which occurs 0.054 radians or $\sim 1.13$ (normalized to an 11 year cycle) months later.

### Accuracy of the clock

This analysis is based on relative occurrence times between the AR areas and *aa* index records. Timings have been compared via the Hilbert transform phase obtained from the monthly SSN record. The accuracy of these comparisons is then limited by the time resolution of the data records and quantities extracted from them. The AR areas are in 50 latitude bins which are uniformly distributed in sin(latitude) and are reported per Carrington rotation. The *aa* index is of 3 h time resolution, and to compare the amplitude of events, we identify the central time of the Carrington rotations during which *aa* exceeds a threshold value of $100-600$ nT. To compare 27 day recurrences, we identify the central time of the Carrington rotations during which the 27 day lagged autocovariance of the *aa* index exceeds 0.25, the autocovariance is estimated over 100 day window. The clock phase zero is set at the occurrence of the average of the last 25 solar minima as times by SILSO, the individual SILSO minima have a STD of 0.3 radians in phase, or 0.5 normalized years, about this value. The switch-on and off are directly related to the averaged mimumum. The switch-on closely coincides with the average phase of the last 12 terminators^37^, which occurs 0.054 radians or $\sim 1.13$ (normalized to an 11 year cycle) months later. The individual terminators also have inter-cycle variability and a Hale cycle dependence^25^, so that we do not use their Schwabe cycle average to directly determine the switch-off/on. The stability of absolute clock timings against variation in the choice of smoothing window for the Hilbert transform have been investigated previously^13,51^.

### Extended cycle

A simple model for the latitude of the extended cycle is obtained from the AR centroids as shown in Supplemental Fig. SI3. The Figure plots the AR centroid latitude $\theta$ at the Hilbert phase $\phi$ at the mid point of each Carrington rotation (black points). The AR centroids from multiple solar cycles can be seen to fall on a single path in latitude and phase, recovering the result of Hathaway^52^ that required detailed parametric fitting to the shape of the AR latitude timeseries. A linear least squares regression is obtained for the AR centroids for the most active half of the (normalized) cycle, from the switch on at $+ 2 \pi /5$ to half a cycle later at $2 \pi /5 + \pi$ (overplotted blue points). This avoids fitting to lower latitudes where there is a zone of avoidance known as Spörer’s law of zones^4^. The fitted line (red) is $\theta =a (\phi -b)$ with $a=-3.214$ and $b=7.684$. This fitted line is then extrapolated to give an extended cycle latitude centroid, shown by repeating the AR centroids over a second interval of $2 \pi$ in phase (grey points). The simple model extended cycle terminates (intersects zero latitude) approximately at the switch-on, $2 \pi /5$ phase.

### Differential rotation

Using the ‘standard’ differential rotation curve^30^ for the rotation rate at latitude $\phi$ of $2.90-0.35[sin^2 \phi +sin^4 \phi ]$ μ $Rad s^{-1}$, the differential rotation changes by about 13 % between the solar equator and 15° latitude.

### Identifying 27 day recurrence in the *aa* index

Sargent^22,23^ originally obtained the cross correlation coefficients between successive 27 day intervals of the *aa* index^11,16^ and then performed truncation and smoothing to produce the original R27 index. Here the autocovariance of the *aa* index is used directly as in^25^. For a real-valued discrete signal $x_i$ the raw ($R_m$) and normalized (*acv*(*m*)) covariance^53^ of a sequence with itself (i.e. the “autocovariance”) as a function of lag *m* is, for $m \ge 0$:

1 $$\begin{aligned} R_m= & {} \sum _{n=0}^{W-m-1}\left( x_{n+m}-\frac{1}{W} \sum _{i=0}^{W-1}x_i \right) \left( x_n-\frac{1}{W} \sum _{i=0}^{W-1}x_i \right) \end{aligned}$$

2 $$\begin{aligned} acv(m)= & {} \frac{R_m}{R_0} \end{aligned}$$

with the symmetry property that for $$m < 0$$, $$acv(m)=acv(-m)$$. In the above, the autocovariance is obtained for the sample window $$i=1 \ldots W$$ of the $$x_i$$. The autocovariance of the full resolution *aa* index is calculated at all lags *m* for a $$W=100 $$, 100 day window centred on each day of the record. The 27 day recurrence is considered significant if it exceeds a threshold of 0.25 as this is significantly above the level seen at other lags where recurrence is not expected (10 day lag is plotted in Fig. 2 for comparison). Figure 3f then plots all Carrington rotations within which R(27 days) both exceeds the threshold of 0.25 and the *aa* exceeds a threshold [100 200 300 400 500 600] used throughout.

### Supplementary Information

Supplementary Figures.

## Data Availability

All data used in this study is freely available from the following sources (accessed on 1st October 2023). SILSO Royal Observatory of Belgium, Brussels monthly total sunspot number version 2.0 from 1749: http://www.sidc.be/silso/home. The dates of solar cycle maxima and minima are as determined from the smoothed sunspot number record by SILSO: http://www.sidc.be/silso/cyclesmm. The active region areas record reports total sunspot area (in units of millionths of a hemisphere) found in 50 latitude bins distributed uniformly in Sine(latitude) per Carrington rotation since 1874, available from: http://solarcyclescience.com/AR_Database/bflydata.txt. The 3 h time resolution *aa* index dataset since 1868 is available from the International Service of Geomagnetic Indices at: http://isgi.unistra.fr/. The solar radio flux at 10.7 cm (the F10.7 index) is available since 1947 at: https://www.spaceweather.gc.ca/solarflux/sx-en.php.
