# Supplementary material for: A solar cycle clock for extreme space weather
Source: Sci Rep. 2024 Apr 8;14:8249. doi: 10.1038/s41598-024-58960-5 (PMC11001994; doi:10.1038/s41598-024-58960-5)
Supplement: Supplementary file 1 — Supplementary Figures. [file 41598_2024_58960_MOESM1_ESM.pdf]

Supplementary Information: A Solar Cycle Clock for Extreme Space Weather

Sandra Chapman[1,2,3,*] **and Thierry Dudok de Wit**[2,4]

[1]Centre for Fusion, Space and Astrophysics, Physics Department, University of Warwick, UK
[2]International Space Science Institute, Bern, Switzerland
[3]Department of Physics and Statistics, University of Tromso, Norway
[4]LPC2E, University of Orléans/CNRS/CNES, France
[*]S.C.Chapman@warwick.ac.uk

Supplementary Figures

Figures are as follows:

1. **Figure SI1**: Shows the procedure for performing the Hilbert transform of the SSN record to construct the solar cycle clock, and how the quiet intervals are identified.
2. **Figure SI2**: The AR butterfly diagram of sunspot active region areas versus latitude, areas colour-coded with the occurrence of space weather extreme events.
3. **Figure SI3**: A simple model for the extended cycle path in latitude versus phase of the clock, obtained from the AR latitude centroids.
4. **Figure SI4**: Sunspot AR latitudes colour-coded with extreme and recurrent geomagnetic activity plotted versus time for the last 13 solar cycles. These are overplotted with the latitudes of the upper bound, and the centroid, latitudes of the AR areas, to provide a time-unfolded version of Figure 3 main paper.

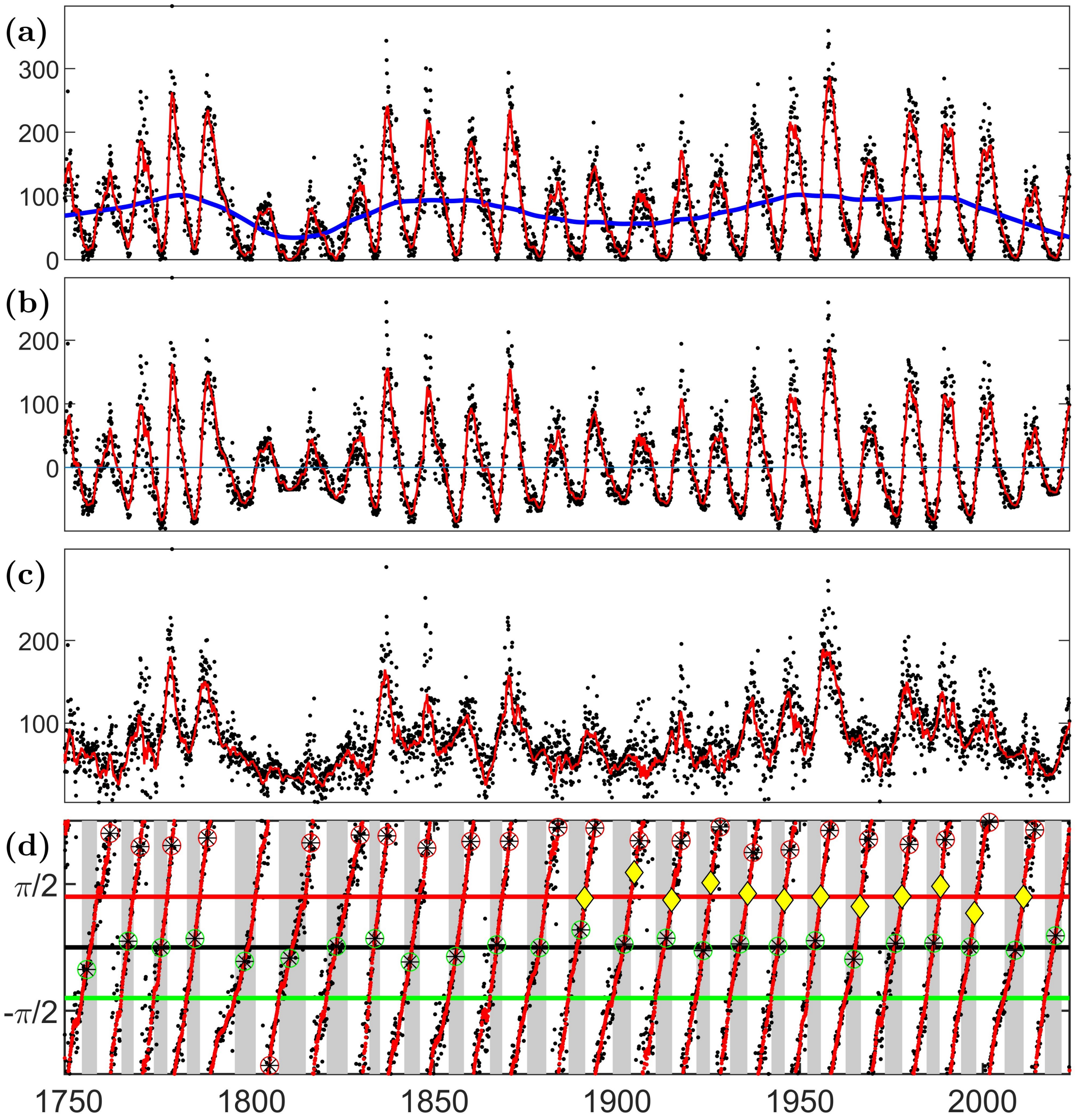

Supplementary Figure SI 1. Constructing the solar clock and identifying the quiet intervals of the solar cycle. The Figure shows the procedure for performing the Hilbert transform of the SSN record. Panel (a): the monthly SSN (black dots), its 13 month running mean (red) and 40 year rlowess slow trend (blue). Panel (b) Monthly SSN and its 13 month running mean with slow trend subtracted. The time traces in panel (b) are Hilbert transformed and the analytic amplitude and phase are plotted in panels (c) and (d) respectively. Panel (d): the solar cycle maxima and minima (catalogued by SILSO) are indicated by red and green circled black asterisks respectively. Zero phase is set at the average phase of the minima of cycles 1-25. Yellow diamonds indicate terminators. The switch-off (green line) and on (red line) are at phases $\pm 2\pi/5$ either side of the averaged minimum. The switch-off and switch-on phases then map to switch-off and switch-on times for each solar cycle which define the quiet intervals of each cycle (grey shading).

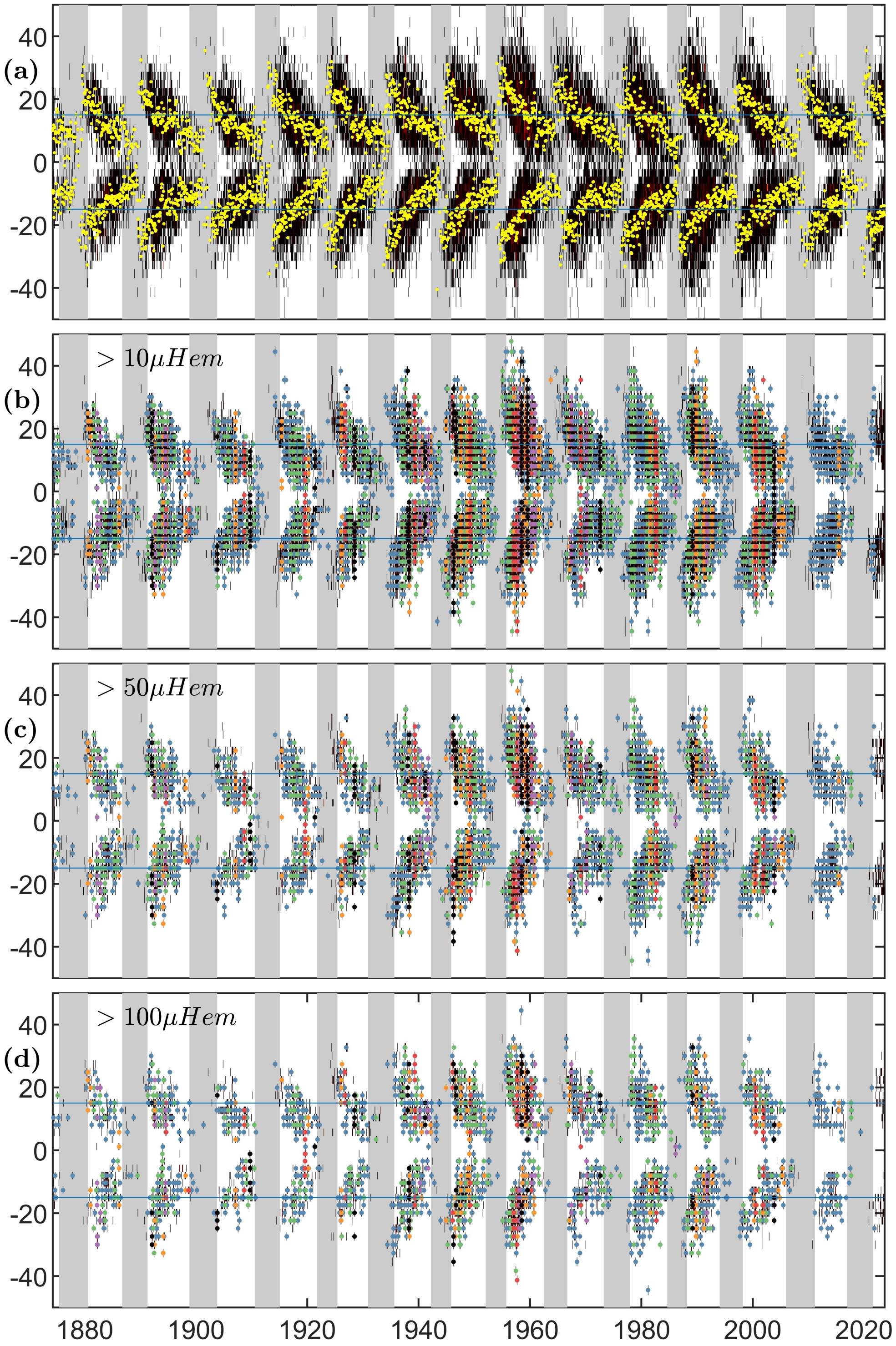

Supplementary Figure SI 2. Butterfly diagram of sunspot areas colour-coded with space weather extreme events. Panel (a): total sunspot area (in units of millionths of a hemisphere, μHem) found in 50 latitude bins distributed uniformly in Sine(latitude) per Carrington rotation, overlotted (yellow) the area centroid for each Carrington rotation. Panels (b-d) plot total sunspot areas in bins where the area exceeds 10, 50 and 100 μHem respectively, overplotted are these bins colour-coded when the *aa* geomagntic index exceeds a threshold of 100 (blue) 200 (green) 300 (orange) 400 (red) 500 (purple) 600 (black) nT. Horizontal blue lines indicate latitudes $\pm 15^{\circ}$.

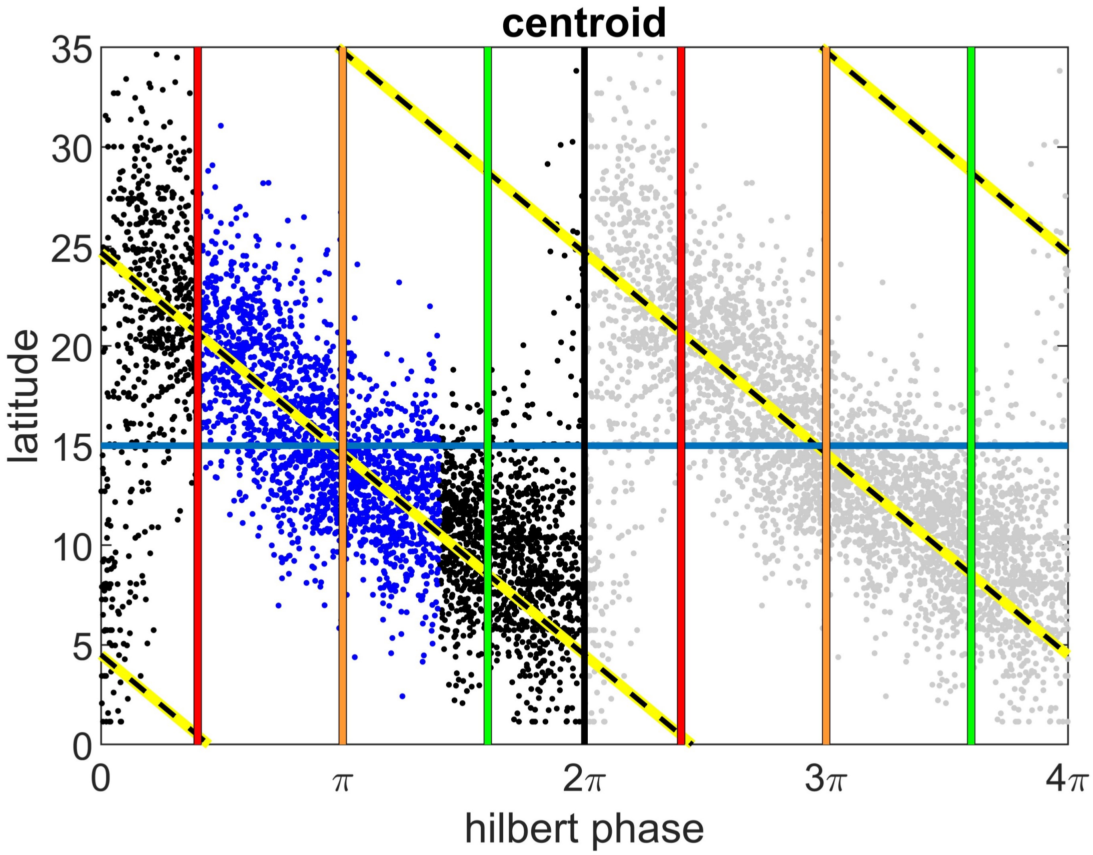

Supplementary Figure SI 3. Simple model for the extended cycle. A simple model for the latitude of the extended cycle is obtained from the AR centroids. The Figure plots the AR centroid latitude θ at the Hilbert phase ϕ at the mid point of each Carrington rotation (black points). The model parameters are obtained from the linear least squares regression for the most active half of the (normalized) cycle, from the switch on at $+2\pi/5$ to half a cycle later at $2\pi/5+\pi$ (overplotted blue points). The resulting fit (yellow highlighed black dashed lines) is $\theta = a(\phi - b)$ with $a = -3.214$ and $b = 7.684$, it is extrapolated to model the extended cycle, shown by repeating the AR centroids over a second interval of 2π in phase (grey points). The simple model extended cycle terminates (intersects zero latitude) approximately at the switch-on, $2\pi/5$ phase, and crosses 15° latitude (horizontal blue line) at π phase. The switch-on and off are indicated by vertical red and green lines respectively, the plot is centred on the average minimum which is at zero (2π) phase (black line).

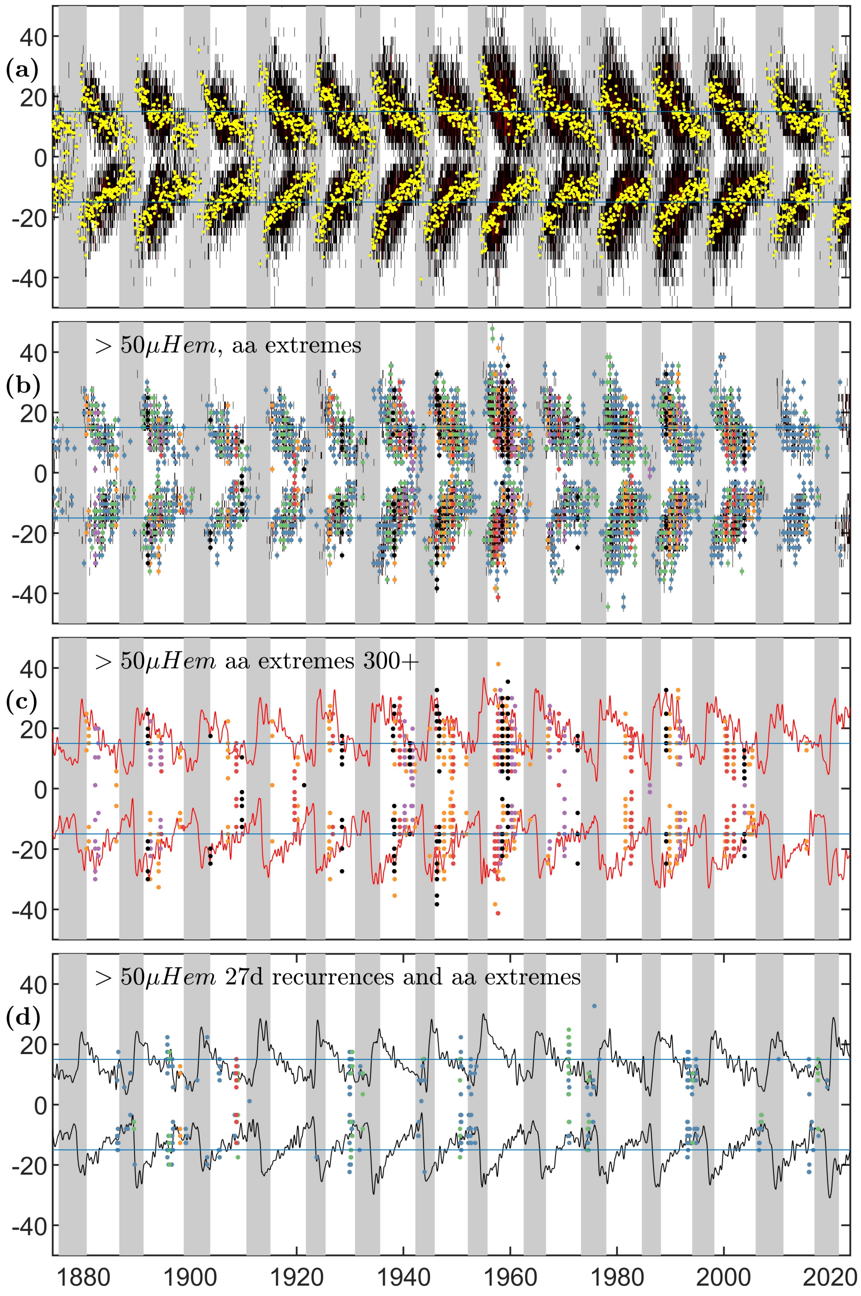

Supplementary Figure SI 4. Sunspot area latitudes and extreme and recurrent geomagnetic activity overplotted for the last 13 solar cycles. All panels: latitude plotted as a function of time. Blue horizontal lines plot latitude $\pm 15°$. The quiet interval between the switch-off and in indicated by grey shading. Panel (a) total sunspot area in single Carrington rotation-latitude bins (black) overplotted with the area centroid for each Carrington rotation (yellow). Panel (b) overplots on all sunspot area latitude bins with area $> 50\mu Hem$ at the Carrington rotation during which the *aa* index exceeds a threshold of 100 (blue) 200 (green) 300 (orange) 400 (red) 500 (purple) 600 (black) nT. Panel (c) plots at all latitude bin centroids at the Carrington rotation during which the *aa* index exceeds a threshold of 300 (orange) 400 (red) 500 (purple) 600 (black) nT. The red lines plot 13 Carrington rotation smoothed latitudes below which 90% of total sunspot AR area lie. Panel (d) as for panel (b) for Carrington rotations where the *aa* index both exceeds the threshold and has a 27 day lag autocovariance exceeding 0.25. The black lines plot 20 Carrington rotation smoothed sunspot areas latitude centroids.
